# Management of complications of mega-implants following treatment of primary and periprosthetic fractures of the lower extremities

**DOI:** 10.1038/s41598-023-44992-w

**Published:** 2023-10-16

**Authors:** M. Ghanem, A. Kalb, C.-E. Heyde, A. Roth

**Affiliations:** 1 Department of Orthopedics, Traumatology and Plastic Surgery, University Hospital Leipzig (Klinik und Poliklinik für Orthopädie, Unfallchirurgie und Plastische Chirurgie, Universitätsklinikum Leipzig AöR), Liebigstrasse 20, 04103 Leipzig, Germany; 2 Department of Physical Therapy and Rehabilitation, University Hospital Leipzig, Leipzig, Germany

**Keywords:** Diseases, Trauma

## Abstract

In recent years, indications for implanting mega-implants were established in managing major bone defects linked to revision arthroplasty due to loosening, periprosthetic fractures, re-implantation following periprosthetic joint infection, non-union following fractures as well as complex intraarticular primary fractures. This study was conducted to discuss and analyze the strategy of diagnosis and management of complications following the use of mega-implants in treating primary and periprosthetic fractures of the lower extremities. This is a monocentric retrospective study. Patients aged ≥ 18 years who underwent implantation of a megaendoprosthesis due to periprosthetic or primary fractures of the lower extremity between January 2010 and February 2023 were identified from the authors’ hospital information system. We identified 96 patients with equal numbers of fractures (71 periprosthetic fractures and 25 primary fractures). 90 cases out of 96 were investigated in this study. The drop-out rate was 6.25% (six cases). The average follow-up period was 22 months (1 to 8 years) with a minimum follow-up of 1 year. The diagnosis of complications was provided on the basis of subjective symptoms, clinical signs, radiological findings and laboratory investigations such as C-reactive protein, leucocyte count and the microbiological findings. The indications for implantations of modular mega-implants of the lower extremities were periprosthetic fractures (65 cases/72.22%) and primary fractures (25 cases/27.78%). Pathological fractures due to malignancy were encountered in 23 cases (25.56%), in one case due to primary tumor (1.11%) and 22 cases due to metastatic lesions (24.44%). Two cases (2.22%) presented with primary intraarticular fractures with severe osteoporosis and primary arthrosis. In all cases with malignancy staging was performed. Regarding localization, proximal femur replacement was encountered in 60 cases (66.67%), followed by distal femur replacement (28 cases/31.11%) and total femur replacement (2 cases/2.22%). The overall complication rate was 23.33% (21 complications in 21 patients). The most common complication was dislocation which was encountered in nine cases (10%), all following proximal femoral replacement (9 cases out of 60, making 15% of cases with proximal femoral replacement). The second most common complication was infection (six cases, 6.67%), followed by four aseptic loosenings (4.44%), further intraoperative periprosthetic fracture in one case (1.11%) and a broken implant in one case (1.11%). We noticed no cases with wear and tear of the polyethylene components and no cases of disconnections of the modular components. Mega-endoprostheses enable versatile management options in the treatment of primary and periprosthetic fractures of the lower extremities. The rate of complications such as loosening, implant failure, dislocation and infection are within an acceptable range in this preliminary analysis. However, implantation of mega-endoprostheses must be strictly indicated due the limited salvage options following surgery.

## Introduction

The initial clinical experience with modular mega-implants was linked to tumor surgery of the musculoskeletal system^38^. In recent years, mega-endoprostheses have gained increasing significance and wider use in revision arthroplasty of hip and knee joints^11,38^. Over this period, indications for implantation of mega-implants were established in managing major bone defects linked to revision arthroplasty due to loosening, periprosthetic fractures, re-implantation following periprosthetic joint infection, non-union following fractures^2,9,11,40^ as well as complex intraarticular primary fractures^39^. The vast majority of mega-implant systems consist of modular components. The use of modularity offers improved versatility and reconstruction options^2,40^. However, surgical interventions involving mega-implants are major surgeries that are associated with high complication rates^2,16^. Additionally, the use of mega-implants naturally restricts the options for managing potential postoperative complications^2,11,38^.

Dislocation is the most common postoperative complication after revision arthroplasty of the hip joint using mega-endoprostheses^29^. The loss of the dynamic stabilization of the muscular cuff of the hip is the most common cause of high rates of dislocation^36^. The use of a dual mobility acetabular cup reduced the rate of dislocation^3,8,12^.

Nevertheless, infection is a serious complication of revision arthroplasty when mega-implants are used in hip and knee surgery with an average documented rate of 18%, ranging between 3 and 36%^10,13,15,19,27,33,40,41^. Most cases of infection reported in studies were recurrent infections following two-stage surgical treatment of the initial periprosthetic infection^2,11,38^.

The purpose of this study was to discuss and analyze the strategy of diagnosis and management of complications following the use of mega-implants in treating primary and periprosthetic fractures of the lower extremities.

## Methods

### Study design

The study was completely carried out at the department of orthopaedics, traumatology and plastic surgery, university hospital of Leipzig. It was approved by the ethics committee of the local university. The vote-number of the audit authority is 020/21-ek.

Patients aged $\ge$ 18 years who underwent implantation of a megaendoprosthesis due to periprosthetic or primary fractures of the hip and knee between January 2010 and February 2023 were identified from the authors’ hospital information system. For all patients who were identified as eligible, a retrospective chart review was performed. Patients with periprosthetic femoral fractures as well as patients with pathological fractures of the femur that were treated by proximal, diaphyseal, distal or total femur replacement were included. Periprosthetic proximal femoral fractures were classified according to the Vancouver classification published by Duncan and Masri^6^, periprosthetic distal femoral fractures were classified according to Rorabeck and Taylor^30^ and periprosthetic tibial fractures according to Felix et al.^7^. All patients consented to the use of their data for research purposes.

### Data collection

Baseline characteristics and information on hospitalization time, comorbidities, mobility, complications and revision surgery were obtained. We identified 96 patients with equal numbers of fractures (71 periprosthetic fractures and 25 primary fractures). 90 cases out of 96 were investigated in this study. The drop-out rate was 6.25% (six cases), due to death of some patients involved, mostly of cardiovascular diseases. The average follow-up period was 22 months (1 to 8 years) with a minimum follow-up of 1 year. None of the included patients died during the study.

We analyzed patients’ data based on their archived records and electronic files in SAP (SAP A, Walldorf, Germany) as well as radiological findings and images from SIENET MagicWeb/ACOM (Siemens AG Healthcare Sector, Erlangen, Germany).

### Statistical analysis

Statistical analysis was performed by using the spreadsheet software Microsoft Excel (Microsoft Corporation, Redmond, USA). A Kaplan–Meier survivorship analysis was conducted^23^.

### Implant-related information

In 88 cases we used the München-Lübeck™ modular endoprosthesis system (AQ Implants, Ahrensburg, Germany, currently AQ-Solutions GmbH, Hürth, Germany). In 2 cases concerning the knee joint we used the Revision LPS Insert with S-ROM® NOILES™ Rotating Hinge System and the LPS System Surgical Technique DePuy Synthes (325 Paramount Drive, Raynham, MA, USA). Both modular mega-implant systems consist of extraosseous modules for the hip, knee and bone shaft and intramedullary stems. The individual components are connected by conical clamping and secured by a locking screw. The selection and combination of individual modular implant components of this system allows intraoperative adjustment of length, rotation and curvature.

Acetabular cups of the Revisio® M (AQ-Implants, Ahrensburg, Germany, currently AQ-Solutions GmbH, Hürth, Germany) were used. In case of dual mobility cups, the BiMentum™ dual mobility cup Surgical Technique DePuy Synthes (325 Paramount Drive, Raynham, MA, USA) was used. Highly cross-linked polyethylene liners were used in all cases. For the hip joint, ceramic heads were used in patients under 80 years, metal heads in patients over 80. In cases not requiring acetabular replacement, the Self Centering™ Bipolar Head DePuy Synthes (325 Paramount Drive, Raynham, MA, USA) was used.

Taking the femoral antecurvation into account, curved femoral medullary stems were used, whenever possible. The München-Lübeck™ modular endoprosthesis system comprises of 120 mm or 160 mm cemetable stems, the 120 mm stems are straight, the 160 mm are slightly curved. Both have a variety of diameters. The modular parts comprise of trochanter modules, condylar modules as well as extension modules 30 mm to 120 mm.

The Revision LPS Insert with S-ROM® NOILES™ Rotating Hinge System and the LPS System Surgical Technique DePuy Synthes was used in two cases with replacement of the knee and distal femur. The system provides cementable and cementless implants. In the two cases we used cemetless implants due to the relative young age of the patients and the sound bone structure. The cemetless system relies on metaphyseal and adjacent diaphyseal anchoring through the sleeves (see Fig. 5). Intramedullary stems are straight and 75 mm, 115 mm or 150 mm long with a variety of diameters.

### Surgical technique

All surgeries were performed with the patient in the supine position. We used the anterolateral approach for the hip joint, the lateral approach for the midshaft of the femur and a median incision for the knee joint with medial joint approach. In cases of total replacement of femur, two approaches have been performed; the anterolateral approach of the hip joint was extended to the lateral aspect of the proximal two thirds of the femur and the knee joint was approached as mentioned above. In all cases of proximal femoral replacement, reconstruction of the pelvitrochanteric musculature was attempted fixation to the musculus vastus lateralis. No synthetic fiber tissue band were used.

### Diagnosis of complications

The diagnosis of complications was provided on the basis of subjective symptoms, clinical signs, radiological findings and laboratory investigations such as C-reactive protein, leucocytic count and the isolation of microbes. Infection was diagnosed according to the Musculoskeletal Infection Society criteria for periprosthetic joint infection^26^.

In cases of dislocation, suspicion is based on clinical presentation and radiographs are used to confirm and asses. Aseptic loosening were diagnosed based on symptoms such as pain on weight bearing, clinical findings including limping and reduced range of motion, though these signs are non-specific, and simple two-plane x-ray imaging. In these cases, infection could be excluded based on non-pathological laboratory findings and lack of evidence of pathogens in punctures which were performed in each of the four cases.

### Ethical approval

Approval of the local institutional review board for study had been given (Ethical Committee at the Medical Faculty, Leipzig University, AZ 020/21-ek) in view of the retrospective nature of the study and all the procedures being performed were part of the routine care.

### Consent to participate

All individuals have given general consent in the use of their data, including imaging, for analysis and publication. This has been approved by the Ethical Committee. Informed consent was obtained from all subjects and/or their legal guardian under Ethical approval and consent to participate section.

### Consent for publication

All individuals have given general consent in the use of their data, including imaging, for analysis and publication. This has been approved by the Ethical Committee.

## Results

### Patient cohort

The patient cohort comprised 51 females (56,67%) and 39 males (43.33%). Their average age at the time of surgery was 76 years (58–92 years). 47 of the implants were on the right side (52.22%) and 43 on the left side (47.78%). Simultaneous bilateral implantation was not performed in any of the cases.

### Indications for surgery

**Figure 1:**
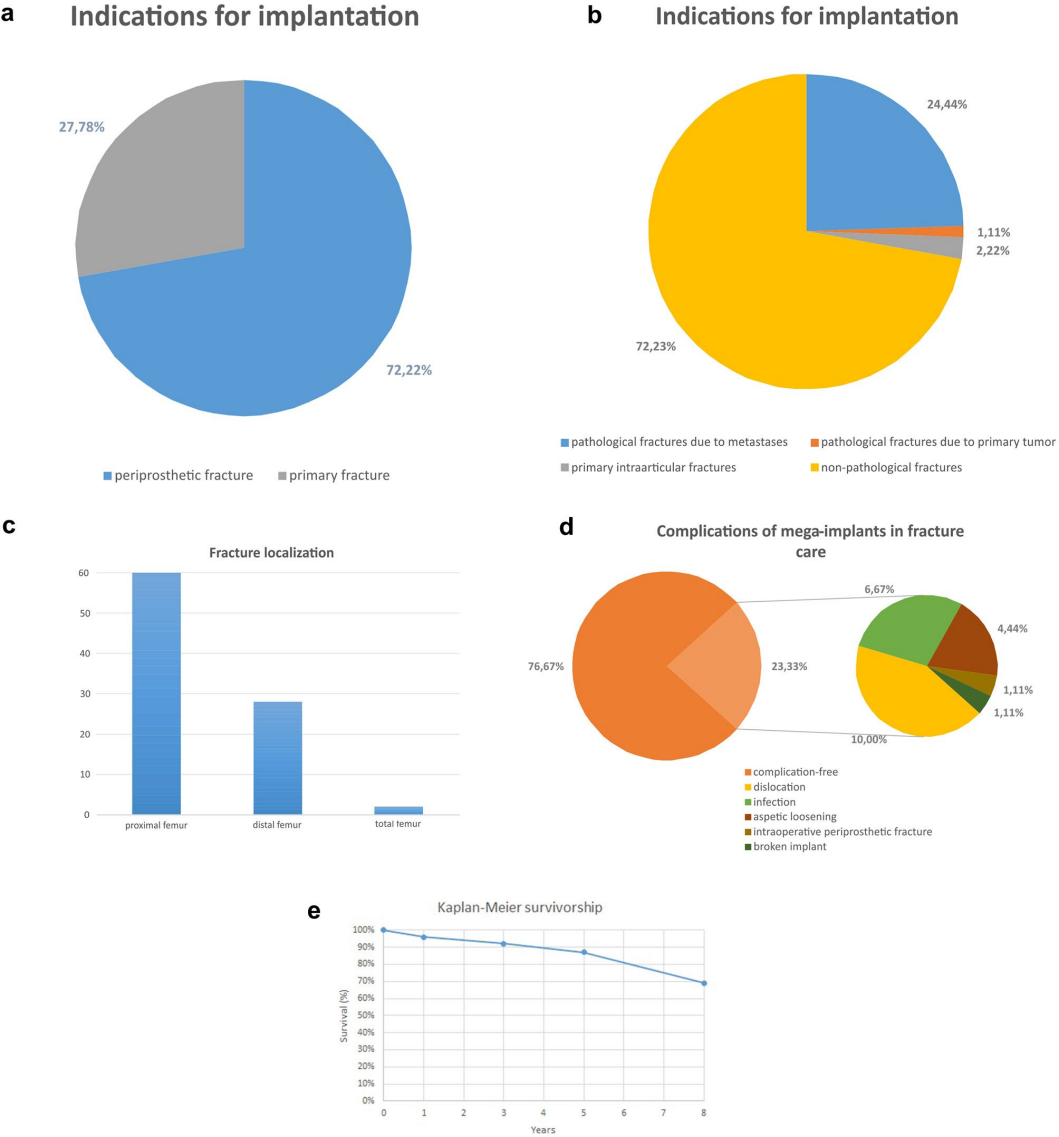
(**a**) and (**b**) Indication for implantation of mega-implants in treatment of primary and periprosthetic fractures of the lower extremities. (**c**) Localization of femur replacement in the treatment of fractures of the lower extremities in absolute numbers. (**d**) Complication rate of mega-implants in fracture care. Out of 90 cases 76,67% were complication free, leaving 21 cases (23,33%) with complications as shown as above. The most common complication was dislocation, followed by infection and aseptic loosening. (**e**) Kaplan–Meier survivorship free of any revision.

The indications for implantations of modular mega-implants of the lower extremities (Fig. 1a) were periprosthetic fractures (65 cases/72.22%) and primary fractures (25 cases/27.78%). The 65 cases with periprosthetic fractures included 40 cases with Vancouver B3-fractures, 24 distal femoral fractures Type III according to Rorabeck and Taylor and one periprosthetic proximal tibial fracture Type IIC according to Felix et al.

**Figure 2:**
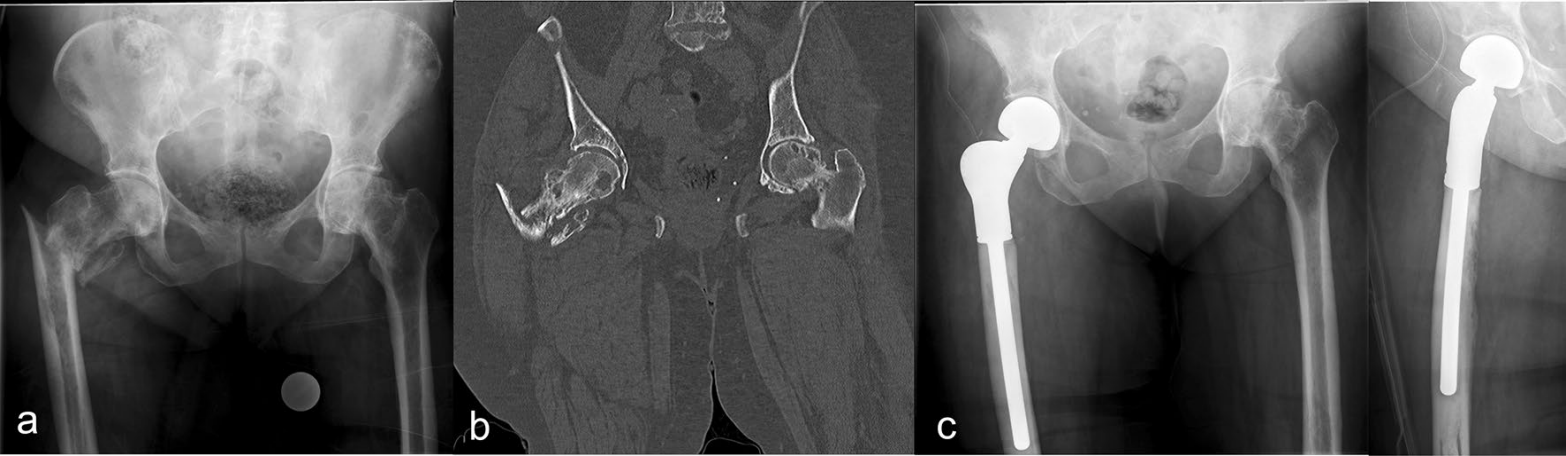
A pathological femoral fracture due to metastatic breast cancer, shown on preoperatively x-ray (**a**) and CT-scan (**b**), that was treated with a proximal femoral replacement (**c**).

**Figure 3:**
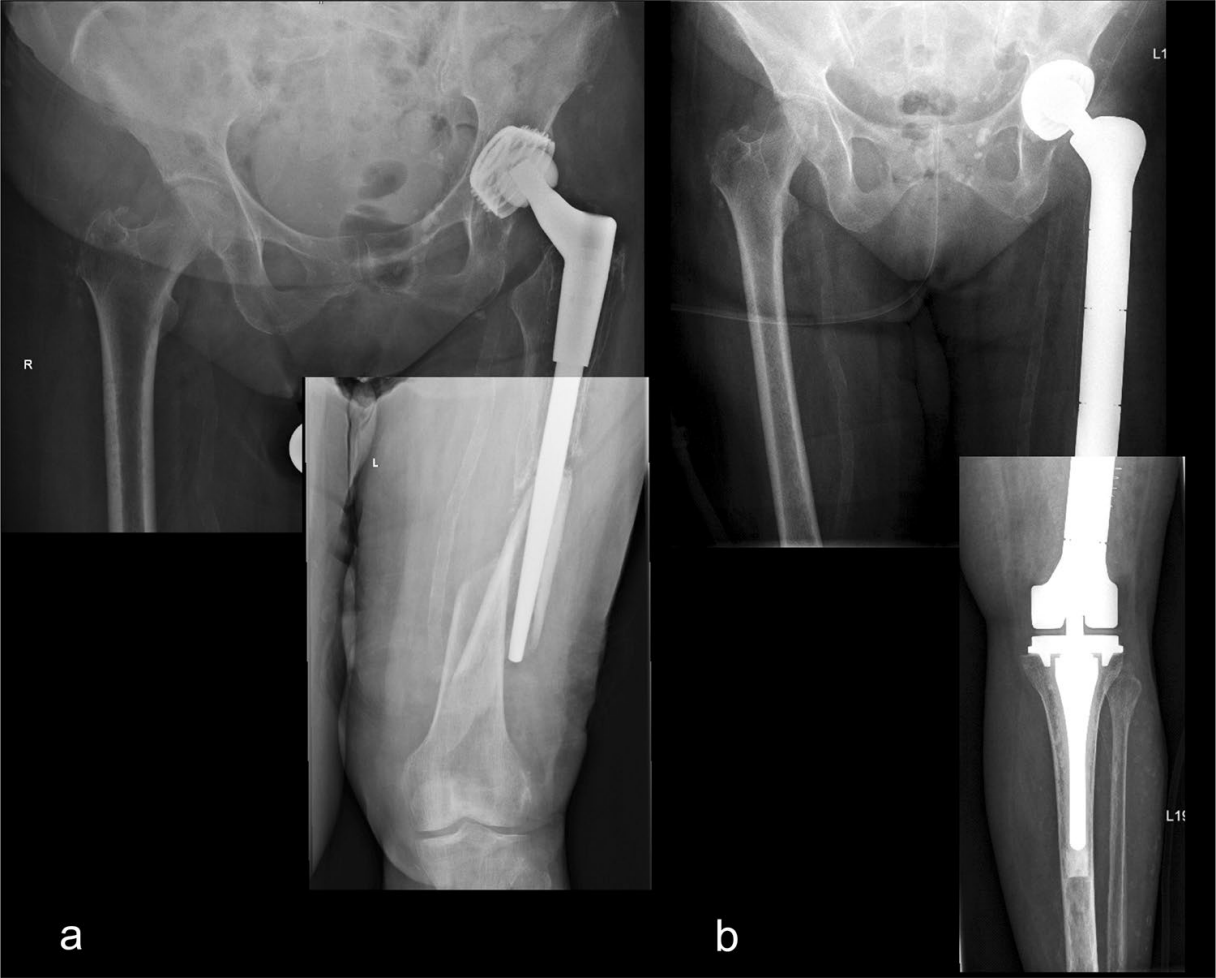
Preoperative (**a**) and postoperative x-rays (**b**) of a total femoral replacement after recurrent periprosthetic fracture following a total hip replacement in a female patient with multiple comorbidities. A swift performance of surgery was necessary.

Primary pathological fractures due to malignancy were encountered in 23 cases (25.56%), in one case due to primary tumor (1,11%) and 22 cases due to metastatic lesions (24.44%) (Fig. 2). The primary tumor was osteosarcoma, which surgically resected in sano and treated via neo-adjuvant chemotherapy. The five-year follow-up revealed no recurrence. Two cases (2.22%) presented with primary intraarticular fractures with severe osteoporosis and primary osteoarthritis (Fig. 1b). Regarding the affected joints (Fig. 1c), proximal femur replacement was encountered in 60 cases (66.67%), followed by distal femur replacement (28 cases/31.11%) and total femur replacement (2 cases/2.22%) (Fig. 3).

### Complications

The overall complication rate was 23.33% (21 complications in 21 patients). The most common complication (Fig. 1d) was dislocation which was encountered in nine of all cases (10%), all following proximal femoral replacement 15% of cases with proximal femoral replacement). The second most common complication was infection (six cases, 6.67%), followed by four cases with aseptic loosening (4.44%), further intraoperative periprosthetic fracture in one case (1.11%) and a broken implant in one case (1.11%). We noticed no cases with worn polyethylene components and no cases of disconnections of the modular components.

The Kaplan–Meier survivorship free of any revision was 96% at one year, 87% at five years and 69% at eight years (Fig. 1f).

Out of the nine dislocations, we successfully performed three closed reductions and supplied the patients with orthoses with complete limitation of adduction and limitation of flexion of the hip joint to 45 degrees for a period of six weeks, with no subsequent recurrence of dislocation. In six cases open reduction was necessary. In two of them we replaced the neutral acetabular inlay with an asymmetrical inlay with no subsequent recurrence. Therefore, we implanted a constrained inlay in one case and performed replacement of the acetabular cup with a tripolar acetabular cup system in further three cases with no subsequent recurrence. All dislocations were observed in cases following previous surgeries. In all cases, we encountered a pre-existing insufficiency of the pelvitrochanteric musculature. Acoordingly, reconstruction was significantly limited or technically not possible. Clinical and radiological analyses revealed no significant malposition of the implants.

**Figure 4:**
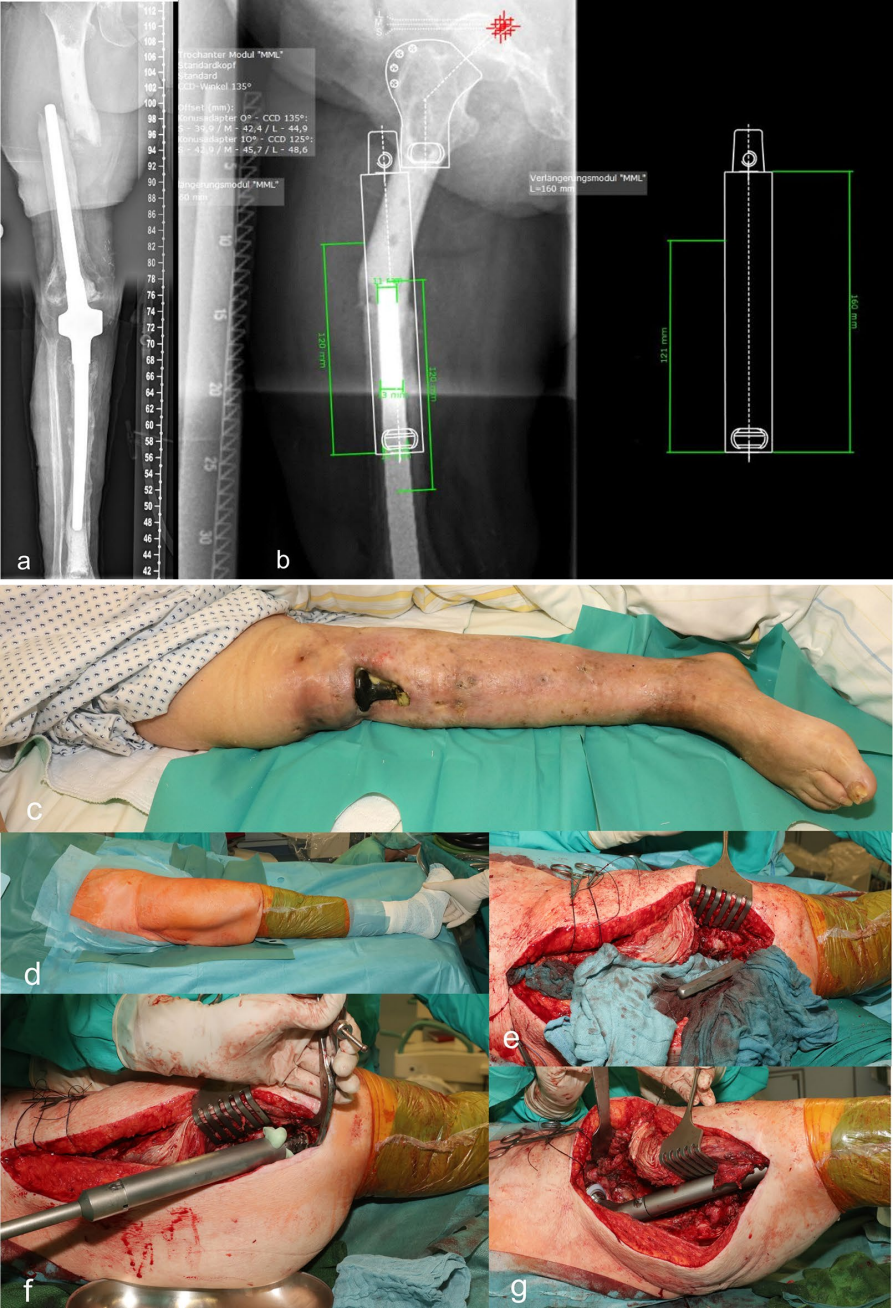
X-ray of a female patient, who presented with a periprosthetic fracture of the proximal femoral shaft (**a**). The patient was suffering from a chronic infection of the intramedullary knee arthrodesis after previous surgeries with a soft tissue defect and longstanding open wound at the ipsilateral knee (**c**). Special preoperative computerized planning for the off-label use of the modular component from a different company was necessary (**b**). Intraoperative pictures of the preparation of the arthrodesis stem (**e**), off-label fitting of the modular component on the fixed stem using bone cement (**f**) and the final reduction in situ (**g**). The side of the infected arthrodesis of the knee was covered at all time (**d**). Postoperative antero-posterior x-ray imaging of the fitted implant after resection and replacement of the proximal femur (**e**). Postoperative clinical findings with a proximal wound after implantation of a proximal femur replacement with no signs of local infection and the existing soft tissue defect of the knee (**f**). On a clinical and radiological check-up more than six months after surgery, the patient presented mobile without the use of crutches for a limited distance in her home with total weight bearing on the right leg. As expected, we saw clinical signs of infection at the proximal implant site as well as a raise in C-reactive protein (**g**).

Two cases with early infection due to staphylococcus aureus were treated and controlled by exchanging polyethylene inlays, performing debridement and lavage with subsequent antibiotic therapy not exceeding six weeks. Further three cases comprised chronic infections with staphylococcus epidermidis that were treated with two-stage surgery with explantation of all components and temporary implantation of cement spacers prior to reimplantation in the second stage. The remaining case is very particular (Fig. 4). In this case, the patient had a longstanding infection of the intramedullary knee arthrodesis after previous surgeries due to initial fracture and subsequent osteomyelitis. The patient first presented to us following a periprosthetic fracture of the proximal femoral shaft. The local extremity board recommended amputation which the patient refused. Replacement of the proximal femur with implants that are compatible with the components of the knee arthrodesis would have necessitated disconnection of the arthrodesis and subsequent coupling of the compatible components with replacement of the proximal femur. This would have entailed the direct spread of infection to the proximal femur and the hip joint. Instead, we offered an off-label use of a modular component from a different company. The surgical procedure was performed without direct exposure of the infected arthrodesis of the knee. More than six months after surgery, the patient did not complain of pain and could walk limited distances without the use of crutches or any other orthopedic aids. Yet, local signs of infection in the hip joint were evident. The patient refused further surgical intervention. In case of deterioration of the local or general condition, the patient would consent to the creation of a stable fistula as a salvage procedure.

The four cases with aseptic loosening were diagnosed after an average period of seven years after implantation of mega-endoprostheses, ranging from 5–10 years. Two cases involved proximal femoral replacement and two cases distal femoral replacement. In all cases the intramedullary stem was replaced using cemented implants.

**Figure 5:**
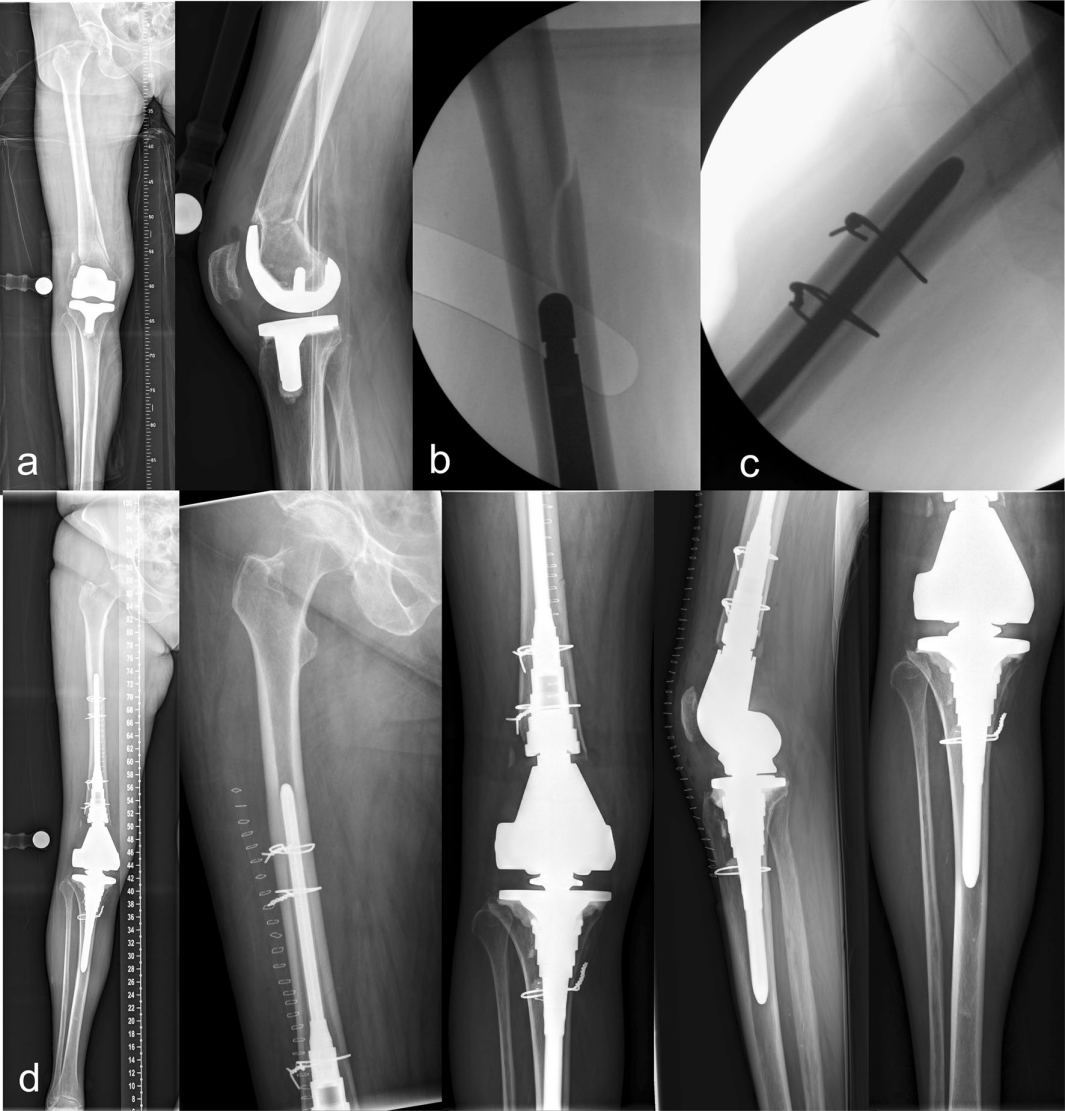
X-rays of an intraoperative periprosthetic fracture of the proximal tibia and proximal femur in a distal femoral replacement (**a**), known by clinical findings and intraoperative x-ray (**b**), which was treated with the implantation of a longer stem and cerclage (**c**) as well as partial weight bearing for six weeks postoperatively (**d**).

An intraoperatively periprosthetic fracture occurred in one case (Fig. 5). In this particular case, the implants with the smallest available dimension were used according to preoperative planning. Despite that, further intraoperative fractures in the proximal tibia was encountered, which was reduced and fixed with a cerclage and a proximal femoral fracture which was managed with a longer intramedullary stem, double cerclage, and postoperative partial weight bearing for six weeks. The complication was diagnosed based on clinical findings and two-plane x-ray imaging. Signs of bone healing were detected radiologically six weeks after which full weight bearing was allowed.

**Figure 6:**
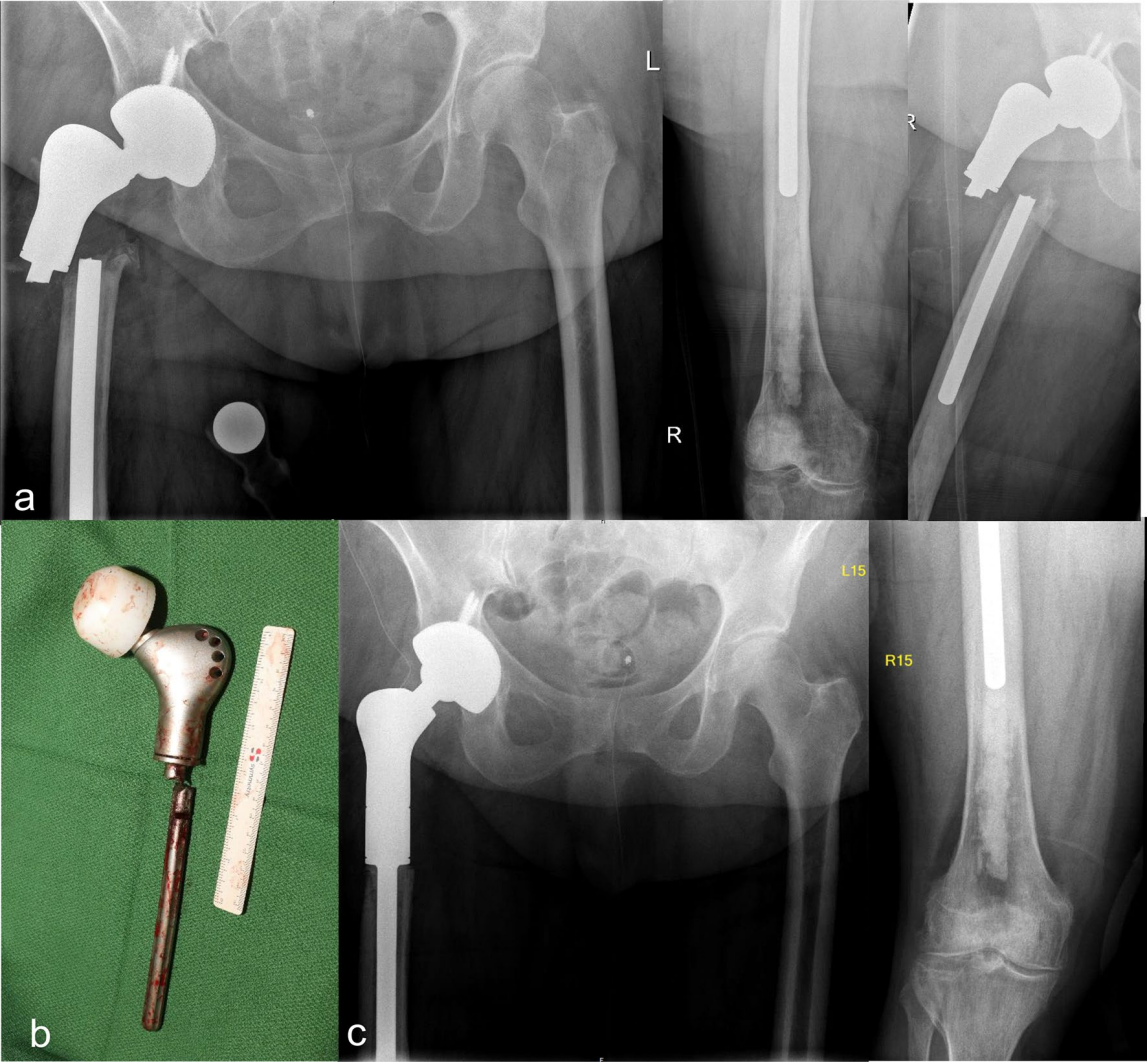
A broken implant (**b**), diagnosed on clinical findings and x-ray imaging (**a**), that was cared for in a single stage replacement with explantation and reimplantation with cement in cement (**c**).

In one case we encountered a broken implant (Fig. 6). Diagnosis was suspected based on clinical findings and confirmed by two-plane x-ray imaging. Treatment was carried out by single-stage replacement with explantation of the stem and reimplantation with cement in cement.

## Discussion

The use of modular mega-implants is associated with a higher perioperative complication rate than in primary arthroplasty^9,28^. Contributing factors are the long duration of surgery and the large bone and soft tissue defects that are associated with an extensive wound area. The wide range of complications that might occur includes both intra- and postoperative complications^9,17,28,38^.

Overall, intraoperative complications such as vascular or organ lesions or periprosthetic fractures are relatively rare and can be directly treated^32^.

Urinary tract infections, pneumonia or cardiovascular and thromboembolic events can also occur during hospitalization^28^.

Postoperative complications can be divided into mechanical and non-mechanical complications, and early and late complications based on their time of occurrence, and based on their etiology^17^. Non-mechanical complications such as periprosthetic infections are serious and require differentiated management^9,11^. Mechanical complications manifest as^17^:

- Aseptic loosening of components (particularly intramedullary shafts),
- Break of implants,
- Disconnection of modular components,
- Dislocation of the hip joint after proximal or total femoral replacement,
- Wear of the components (particularly polyethylene parts) and/or
- Periprosthetic fractures.

The rate of mechanical complications has decreased over time, partly due to technical and material advances. Despite that, aseptic loosening, muscular insufficiency leading to instability and dislocation, periprosthetic fractures or implant breakages are still observed^9,16,17^.

The diagnosis and management of complications following the use of mega-endoprostheses in treatment of primary and periprosthetic fractures of the lower extremities depends on the particular complication. The overall complication rate in our series was 23.33%. The majority of studies report on the rate of specific complications without accurate statements on the overall implant related complication rates. Though, we find overall complication rates in few previously published studies ranging from 27% to 47.8%^1,37^. However, literature reports that 60–84% of the cases with mega-endoprostheses survive five years without the necessity of surgical revision^4,12,14,34^. Consequently, the overall complication rate within the first five years following implantation of mega-endoprostheses of the lower extremities range from 16 to 40%.

While some complications can be treated non-operatively, several revision surgeries are necessary.

The most frequent complication that we encountered in our study was dislocation which was encountered in nine cases (10%), all following proximal femoral replacement (9 cases out of 60, making 15% of cases with proximal femoral replacement). The diagnosis was based on symptoms and clinical signs and was confirmed by conventional x-ray imaging.

Literature also reports on dislocation being the most frequent complication after implantation of mega-endoprostheses^9,16,38^. In the past, high dislocation rates of 25–33% occurred in monopolar socket systems at the hip joint with proximal and total femoral replacement^9,16^. In addition, the difficult soft-tissue refixation and integration in the past were further causes for significant dislocation rates. The dislocation rate was reduced to less than 1–2% after using the duo-head systems and the Trevira connection tape^9,12,16^. Fritzsche et al.^9^ recommend the use of dual mobility cups or tripolar systems. Intraoperatively, particular attention must be paid to avoiding implant-implant or implant-bone impingement. In the case of recurrent dislocations due to axial instability, the use of a snap socket (“constrained liner”) is possible, although this has more secondary complications, such as loosening of the socket, compared to the tripolar system^9^. We did not encounter dislocation in cases treated with bipolar head or with primary acetabular cup replacement using a tripolar system. Therefore, we can confirm the advantage of dual mobility systems.

The second most common complication in our series was infection that we encountered in 6.67%. According to literature, infection is indeed the second most frequent complication encountered after implantation of mega-endoprostheses.

Infection was diagnosed based on symptoms and local clinical signs in addition to laboratory investigations (elevated leucocyte count and C-reactive protein) as well as isolation of microbes in preoperative punctures and/or intraoperative swabs. The authors excluded further infectious foci by performing clinical examination and nuclear medical examination^31^.

Infection rate after implantations of mega-implants ranges from 3 to 36%^5,11,14,16,18,20–22,24,25^.

In accordance with literature, the management of periprosthetic infections depends on the time and severity of the infection, detection of the pathogen with an antibiogram, and the general condition of the patient^2,9,11,20^. In line with literature reports, we regard radical debridement with lavage as the basis for complete infection eradication in combination with resistance-based systemic antimicrobial therapy. In case of an early infection, the implant can be preserved. In case of a chronic infection, all parts of the endoprosthesis must be removed due to the microbial colonization and the mature biofilm. The two-stage replacement of the endoprosthesis shows higher chances of definitive infection eradication^2,9,11,16,38^. An alternative solution is to create a stable fistula as a salvage procedure, especially in patients with comorbidities.

With increasing numbers of cases, the management of periprosthetic infections will continue to represent a major challenge in the future. In addition to an interdisciplinary and patient-specific treatment concept, we also require innovative solutions^11^.

We encountered four cases (4.44%) with aseptic loosening in our series. They were diagnosed after an average of seven years after implantation of mega-endoprostheses, ranging from 5 to 10 years. Aseptic loosening of the endoprosthesis occurs primarily in younger, more active patients due to long-term mechanical stress^9,35^. The loosening rate is reported to be between 5 and 12% and is higher after knee reconstructions than after proximal femur replacements [12; 17; 35]. Movement-dependent pain in the affected region is a typical symptom. The diagnosis includes a conventional X-ray and, if necessary, skeletal scintigraphy. Infection must also be considered in the differential diagnosis. After an infection has been ruled out and depending on the existing bone substance, a one-step stem change can be carried out with or without cement^9^.

Further intraoperative periprosthetic fracture occurred in one case (1.11%) and a broken implant in another case (1.11%).

Periprosthetic fractures and implant fractures are structural complications that occur in about 5% of cases^16^. Implant fractures are rare due to improved material properties^9^. Periprosthetic fractures, on the other hand, can be traumatic or result from aseptic or septic loosening of the endoprosthesis. Therapy depends on localization of the fracture, stability of the shaft anchorage and the existing bone substance. Accordingly, both osteosynthetic procedures and implantation of a longer modular mega-endoprostheses can be considered. In case of infection, the treatment principles for periprosthetic infections are applied^9,11^.

Further, the use of a curved intramedullary femoral stem might prevent further intraoperative periprosthetic fracture.

We noticed no cases with worn polyethylene components and no cases with disconnection of the modular components. Few studies reported on disconnection of modular components of mega-endoprostheses ranging from 0.7 to 3.7%^38,39^.

The limitation of this study lies in its retrospective design, short follow-up period in some cases and a relative inhomogeneity of the patient cohort. We have different implant systems and different indications for using these systems. These multiple variables reduce the statistical power of the study. In addition, no clinical scoring system was carried out. However, this applies to the vast majority of studies that dealt with complications of mega-endoprostheses.

## Conclusion

Mega-endoprostheses enable versatile management options in the treatment of primary and periprosthetic fractures of the lower extremities. The rate of complications such as loosening, implant failure, dislocation and infection are within an acceptable range. However, implantation of mega-endoprostheses must be carefully indicated due the limited salvage options following surgery.

## Data Availability

The datasets used and/or analyzed during the current study are available from the corresponding author on reasonable request.
